# Widefield standing wave microscopy of red blood cell membrane morphology with high temporal resolution

**DOI:** 10.1364/BOE.9.001745

**Published:** 2018-03-16

**Authors:** Peter W. Tinning, Ross Scrimgeour, Gail McConnell

**Affiliations:** 1 Department of Physics, SUPA, University of Strathclyde, Glasgow, G4 ONG, UK; 2 peter.tinning@strath.ac.uk; 3 ross.scrimgeour@strath.ac.uk

**Keywords:** (170.0180) Microscopy, (170.2520) Fluorescence microscopy, (170.3880) Medical and biological imaging, (230.3670) Light-emitting diodes, (100.3020) Image reconstruction-restoration

## Abstract

We report the first demonstration of widefield standing wave (SW) microscopy of fluorescently labelled red blood cells at high speeds that allow for the rapid imaging of membrane deformations. Using existing and custom MATLAB functions, we also present a method to generate 2D and 3D reconstructions of the SW data for improved visualization of the cell. We compare our technique with standard widefield epifluorescence imaging and show that the SW technique not only reveals more topographical information about the specimen but does so without increasing toxicity or the rate of photobleaching and could make this a powerful technique for the diagnosis or study of red blood cell morphology and biomechanical characteristics.

## 1. Introduction

Adult mammalian red blood cells are a unique cell type which lacks a nucleus, mitochondria and other organelles. In most mammalian species red blood cells have a characteristic biconcave shape that seems to permit increased manoeuvrability and faster diffusion of oxygen and carbon dioxide across the plasma membrane [1]. There are diseases which have associated membrane morphology changes which include sickle cell anaemia [2,3], hereditary spherocytosis [4,5], and elliptocytosis [5]. These clear morphological changes between healthy and diseased red blood cells make the use of optical microscopy a powerful tool for research or diagnosis.

Fluorescence microscopy has become an integral and fundamental tool for the life sciences, allowing non-invasive study of subcellular structures to be achieved with high-contrast sensitivity and specificity through the use of labelled antibodies, specific molecular probes and photo-proteins [6–8], though the spatial resolution of the technique is limited by the diffraction nature of light. One method to increase the axial resolution in widefield microscopy is to utilize a technique called standing wave (SW) fluorescence microscopy. The method was first demonstrated in 1986 utilising total internal reflection to obtain the SW but the lateral and axial resolutions were severely limited [9]. Using two coherent counter-propagating waves or a simple first surface reflector, it later proved possible to generate the optical SW with the minimum possible nodal spacings [10,11]. As the anti-nodal planes of the SW occur at locations of maximum intensity, any fluorophores that coincide with these maxima are excited whereas at the nodal planes (locations of zero intensity) they do not fluoresce. Periodically-spaced planes of fluorescence emission are thus generated within the specimen that are perpendicular to the optical axis and are spaced by distances of λ/(2n), where λ is the wavelength of excitation light and n is the refractive index of the medium in which the light is propagating.

The FWHM of the anti-nodal planes is used as the definition of the resolution of this technique as it is the positional uncertainty of where an excited fluorophore is located, and it is equal to λ/(4n) which is half the distance of the anti-nodal spacing.

The theoretical SW microscopy point spread function can be calculated using the following equation [9,12]:

$$PSF=\left[1-\text{cos}\left(Kz\right)\right][\text{sin}\;c(\frac{NA^{2}}{2n\lambda_{em}}z){]}^{2}$$ (1)

where $\lambda_{em}$ is the peak emission wavelength, NA is the numerical aperture of the objective lens, K=(4πn)/λ and z denotes a coordinate along the z axis [13,14]. Depending on the wavelength of excitation, the resolution using SW microscopy can be significantly below the axial diffraction limit.

Amor et al. [15], previously reported the use of confocal laser scanning SW microscopy to image the red cell membrane. By placing the specimen on a mirror at the specimen plane they simultaneously imaged multiple anti-nodal planes to create a contour map of the membrane structure. They were able to achieve this in both healthy and unhealthy red blood cells and clearly observe the topography of the red blood cells biconcave section with an axial resolution on the order of 90 nm though the use of confocal microscopy limited their acquisition time to 40 seconds per frame [15].

Whilst SW microscopy allows the observation of axial and lateral movements in the plasma membrane that cannot be seen using standard widefield epifluorescence microscopy, encoding multiple 3D information in a 2D image can make the visualization and extraction of meaningful data not an inconsiderable task. The ability to extract 3D data could allow for the quantification of the cell membrane flickering and movement as well as extracting topographical information about the red blood cell shape in diseased cells or as it undergoes decay.

We report the first use of widefield SW microscopy of red blood cells at 30.30 Hz which is over 1200 times faster than the previous study, allowing for the observation of membrane deformations in real time. Furthermore, we demonstrate a computational method using a combination of standard image processing techniques and custom functions in MATLAB, as we show in Code 1 [16], that make it possible to extract and quantify the SW anti-nodal plane information to create a 3D reconstruction. We also compared the SW movies of the red blood cells to those imaged using standard widefield epifluorescence microscopy to determine if there was any increase in photo-bleaching or toxicity rates.

## 2. Materials and methods

### 2.1 Fluorescently coated lens specimens

Uncoated silica plano-convex lenses, with a focal length of 30 mm and a diameter of 6 mm (Edmund Optics), were cleaned using deionized water and then blow dried with compressed air to remove any contaminants. We amended the lens preparation protocol described by Amor et al. [15], by replacing the APTMS coating with a solution of 0.01% mass concentration poly-L-lysine in H_2_O (Sigma Aldrich) to allow the binding of 1,1'-Dioctadecyl-3,3,3′,3′-Tetramethylindocarbocyanine Perchlorate (DiI) to the lens surface. The specimens and poly-L-lysine solution were placed on a platform rocker for 45 - 60 minutes to evenly coat the curved surface of the lenses in the solution, after which the lenses were thoroughly washed in deionised H_2_O and blow dried.

We created a fluorescent layer on the lens specimen in order to compare our theoretical and experimental SW anti-nodal spacings and FWHM in the same manner as carried out in the work of Amor et al. [15]. To deposit a monolayer of DiI on the curved surface of the lens specimen, a 30 μM solution was prepared by diluting 560 μL of a 1 mg/mL stock solution of DiI (Invitrogen) in 20 ml of dimethyl sulfoxide (DMSO, Sigma).

We coated the lens specimen with DiI which was also used to label the red blood cells and has been used in extensively in red blood cell membrane studies [15,17,18]. Specimens are labelled through direct application of the dye allowing the two lipophilic hydrocarbon tails to diffuse laterally into the membrane after which it fluoresces brightly and it is reported to not cause toxicity to the specimen [19–21]. We investigated other membrane dyes for use, such as DiO, DiA and Di-8-Anepps, but found these unsuitable as either they were internalised by the red blood cells or photobleached too rapidly for practical use.

The lens specimens were placed in a glass petri dish with the curved surface submerged in the dye solution and gently rocked overnight. The petri dish was wrapped in aluminium foil to prevent photo-damage to the dye during this period. The following day the specimens were washed three times in deionized water, then dried using compressed air and kept out of direct light until imaging.

### 2.2 Red blood cell isolation and staining

Blood specimens were obtained on the day of the experiments by cardiac puncture of a single mouse, which was rendered unconscious through CO_2_ exposure, and the collected blood immediately mixed in a centrifuge tube (Star Labs) with acid citrate dextrose (ACD), an anti-coagulant. The ACD comprised of 1.32 g trisodium citrate (Fisher Scientific), 0.48g citric acid (Arcos Organics) and 1.40 g dextrose (Fisher Scientific) and was made up in 100 ml of distilled water. Cardiac punctures were performed by technical staff from the University of Strathclyde’s Biological Procedures Unit in accordance with UK Home Office guidelines and approved by the University of Strathclyde Ethics Committee. The method for isolating and staining the red blood cells has been adapted from the protocol described by Amor et al. [15], and so will be described only briefly here.

The mouse blood and ACD suspension was spun down at 2000 rpm for 10 minutes with the supernatant removed and the pellet resuspended with 500 µL of 4% bovine serum albumin (BSA) (Sigma) in phosphate-buffered saline (PBS) (Gibco). The 4% BSA in PBS had a refractive index of 1.341 that was measured using an Abbe 60 refractometer (Bellington and Stanley Ltd.) which had been calibrated using methanol and glycerol. This process was repeated another three times so only a suspension of red blood cells remained.

Red blood cells were fluorescently labelled by adding 200 µL of the red blood cell suspension to 790 µL 4% BSA in PBS along with 10 µL of a 1 mg/mL stock solution of DiI. The solution was then incubated at 37 °C for 60 minutes whilst being gently shaken to ensure even distribution of the dye. After this they were spun down and resuspended a further four times to remove all excess dye.

Silver broadband mirrors (Thorlabs) were prepared for imaging by first being thoroughly cleaned in ethanol (purity > 99.8%, Sigma). Then to promote specimen adhesion to the mirrors they were coated with a solution 0.1% mass concentration poly-L-lysine (Sigma) and incubated at 37 °C for 45 – 60 minutes whilst being gently rocked. The preparation was washed with PBS and sterilised using UV light. 5 µL of the red blood cell suspension was pipetted onto the mirrors under a coverslip (VWR, thickness = 1.5) 10 minutes prior to imaging.

### 2.3 Video-rate SW imaging of fluorescently labelled specimens

Video-rate SW imaging of the fluorescently labelled specimens was carried out using an upright epi-fluorescence microscope (BX50, Olympus) using a 10x/0.4 dry objective lens (UPlanSApo, Olympus) for the lens specimens and with a 100x/1.4 oil immersion objective lens (UPlanSApo, Olympus). The lens specimens were imaged in air or with a layer of 4% BSA in PBS between the specimen and the mirror to confirm the anti-nodal spacings and FWHM and that the interference pattern was caused by the SW effect.

Illumination of the specimens was provided by a 550 nm LED (pE-4000, CoolLED) with a bandpass filter (535/50, Olympus) which had a peak wavelength of 548.8 ± 1.5 nm (FWHM – 11.9 ± 1.5 nm). The output spectrum of the LED was measured using a spectrometer (USB2000 spectrometer, OceanOptics). A clean up filter was used to prevent bleed-through to the detector, and the LED was coupled to the microscope using a liquid light guide and collimator (Universal collimator, CoolLED).

An average optical power at the specimen plane of 1.71 ± 0.01 mW was used for these experiments. The power readings were obtained by taking three recordings at the specimen plane under the 100x objective lens using a power meter (Fieldmax II, Coherent) with a thermal head (PM10, Coherent) and a three second integration time.

**Fig. 1:**
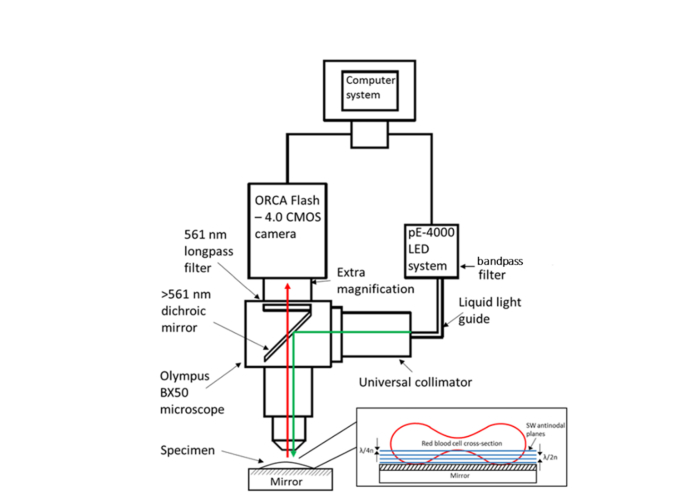
Schematic diagram of experimental setup demonstrating the coupling of the 550 nm LED to the BX50 microscope system via liquid light guide and collimator. The excitation light then reflects of a >561 nm dichroic mirror to generate a SW due to self-interference at the mirror located at the specimen plane. This SW of light causes fluorescence emission at the SW anti-nodes which then propagates upwards through the lens, dichroic mirror and long pass filter to be detected by the CMOS camera. The fluorescence signals are recorded by the computer which synchronizes and triggers the LED and camera.

Fluorescence emission was collected at wavelengths longer than 561 nm using a CMOS camera (ORCA-Flash 4.0LT, Hamamatsu) with a binning n = 2 and an exposure time of 33 ms. Extra magnification was added before the camera to increase the size of the specimen and reduce the number of red blood cells present in the field of view. The extra magnification comprised of an elongated camera mount tube which contained an eyepiece which magnified the images by 2.99x. The LED and camera were synchronized and triggered using the WinFluor imaging and electrophysiology analysis software [22], which also recorded continuous imaging at a rate of 30.30 Hz. Imaging experiments had a duration so as to provide a final SW movie size of 1000 frames. A schematic diagram of the experimental setup can be seen in Fig. 1

.

Using the experimental setup described above we measured a camera pixel size of 44.0 nm. The theoretical widefield resolution according to the Rayleigh criterion for the 550 nm LEDs was calculated to be 239.1 nm laterally and 850.1 nm axially [23]. The lateral resolutions were checked experimentally by imaging 200 nm diameter fluorescent bead mounted in gelvatol, taking a line intensity profile through the centre of the beads and determining the FWHM. Through this process, it was found that the lateral resolution was 290.0 nm. We attribute the difference between experimental and theoretical resolutions to a refractive index mismatch between the immersion oil, gelvatol and the coverslip or perhaps slight aberrations in the optical system. Axial resolution measurements for the SW experiments were checked and compared to the theoretical axial SW resolutions by imaging model fluorescent lens specimens and extracting the anti-nodal plane FWHM and spacings.

### 2.4 Analysis of lens specimen

The SW images of the plano-convex lens specimens were taken to confirm that we were obtaining anti-nodal FWHM and spacings using LED illumination that were comparable to the theoretical values. The images were cropped in ImageJ to only contain eight anti-nodal planes. A MATLAB script was created to analyse the radial signal by taking an average around the centre of the image which reduced the noise and made it easier to extract the anti-nodal spacing and FWHM. This was done using the custom function *radialavg* [24]. The radially averaged intensity was then translated to the axial height from mirror using Pythagoras’s theorem along with the known geometry of the lens specimen with the following equation:

$$d=-(\sqrt{R^{2}-r^{2}})+R$$ (2)

where *R* is the radius of curvature of the lens (15.5 mm) and *r* is the radial distance of each pixel from the centre of the SW image and *d* is the distance from the mirror. To determine the peak intensities for each anti-nodal plane we used the *findpeaks* function to store the positions as a vector. Then by subtracting each anti-nodal position and taking the distance, we obtained the average experimental anti-nodal spacing. The average FWHM values were determined again using the *findpeaks* function then outputting and averaging the width argument from the function.

### 2.5 Algorithm for 3D SW red blood cell reconstruction

All computational analysis and reconstruction was done using MATLAB R2017a on a desktop computer running 64-bit Windows 7 operating system with an 7th generation Intel core i7-770 3.6GHz quad-core processor and 64 Gb of 2400 MHz DDR4 RAM.

The raw SW red blood cell movies were exported from WinFluor and converted to a .TIFF stack. In ImageJ, the images were cropped, and contrast adjusted to only contain the red blood cell of interest. The SW red blood cell images had high frequency noise present due to the instrumentation noise which was reduced by applying a Gaussian blur (MATLAB function *imgaussfilt* with a standard deviation of σ = 2*)* which also preserved the characteristic low frequency anti-nodal and nodal interference pattern of the SW image. Intensity thresholding was then used to extract each of the anti-nodal planes from the background, which was made possible due to the high varying intensity in the images. We utilized a local thresholding method (*adapthresh* set at a 25 by 25 kernel with a sensitivity of 0.60) as the intensity profile was not homogenous across the images. The values for σ and local threshold sensitivity were chosen as they provided the most accurate FWHM and anti-nodal spacings when applied to the f = 30 mm lens specimen and resulted in the highest number of reconstructions when applied to the red blood cell data sets.

The ability to achieve 3D reconstructions of the SW movies was limited by the lateral resolution available with widefield microscopy. As the shape of the red blood cells changed over time, antinodal planes could appear to be in contact with one another, if the separation between planes was less than the lateral resolution of the microscope. To achieve anti-nodal separation, firstly the contrast of the threshold image was inverted such that the nodal regions were represented as ‘1’s and anti-nodal regions were ‘0’s. The background region around the red blood cell was removed such that only the nodal planes were present and using the *bwmorph* function, thinning was applied to the nodal regions to reduce them to one-pixel thick lines. Radial lines were taken at one-degree intervals using the *imrotate* function to store the coordinates of each nodal plane into *n = 3* columns as for the data sets presented here there were only every 3 nodal planes. At the points were there was a discontinuity less than 3 coordinates were detected and had to be assigned to the correct column by comparing it to the last value in each column and determining which was one was numerically closest. In order to fill in the missing column values any column values which were equal to zero were then assigned the value NaN (not a number) which were then replaced by values to obtain a complete nodal plane through cubic interpolation with any outliers removed using the *isoutlier.* The completed planes were then dilated into a 3-pixel thick line using *bwmorph* and subtracted from the original anti-nodal plane threshold image to obtain an image in which all the anti-nodal planes have been separated.

Once the planes were separated we then extracted the boundaries of each of the SW anti-nodal planes using Canny edge detection [25]. The positions of the anti-nodal plane boundaries were indexed, and the values of the x and y Cartesian coordinates stored in a matrix and then each of the anti-nodal planes FWHM were uniquely labelled (*bwlabel*). Utilizing our knowledge of the anti-nodal spacing and the FWHM of each plane we then determined the axial position each lower plane edge ($z_{1}$) and each upper plane edge ($z_{2}$) using the following equations:

$$z_{1}=\frac{3\lambda }{8n}+\frac{\lambda (m-1)}{2n}$$ (3)

$$z_{2}=\frac{\lambda }{8n}+\frac{\lambda (m-1)}{2n}$$ (4)

where m is the plane number above the mirror. Using the *griddata* function, the known x, y and z values where mapped to a 3D surface and the axial positions for all remaining data points were then determined using the cubic interpolation method. By extracting all the intensity values from the antinodal planes in the SW red blood cell images, a custom colormap is created and was assigned to the correct coordinate. The final 3D reconstruction is created using the *scatter3* and s*urf* MATLAB functions and was stored as a .TIFF stack which was converted to an .AVI movie.

## 3. Results

### 3.1 SW imaging of fluorescently coated lens specimens

**Fig. 2:**
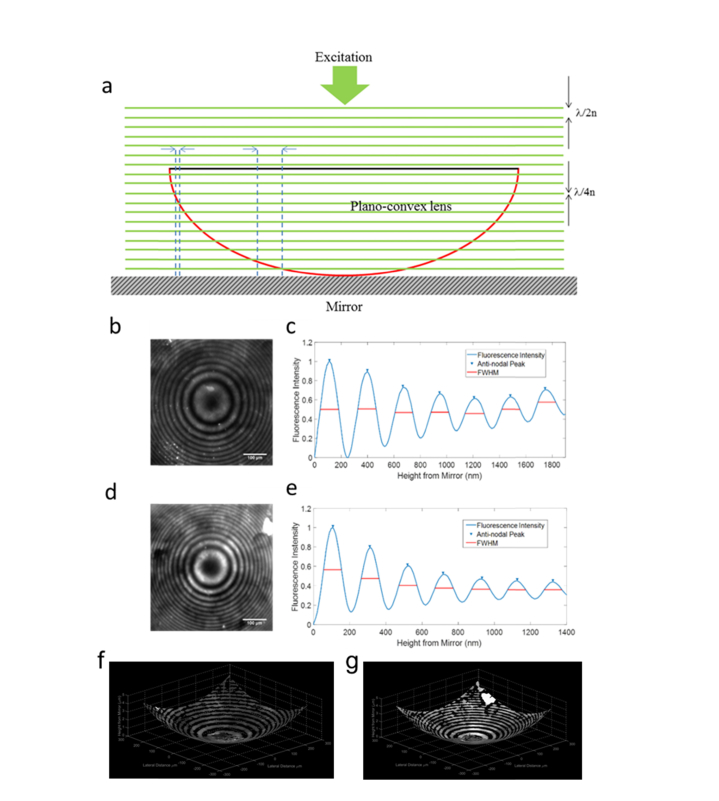
a) A schematic diagram of the fluorescent lens specimen upon the mirror at the specimen plane demonstrating how the SW interacts with the fluorescent coating. Images of the 30 mm focal length lens specimen imaged in air and 4% BSA in PBS are shown in b) and d) respectively. The radial averaged plots of the anti-nodal axial locations of b) and d) are shown in in c), and e). Corresponding 3D reconstructions of each lens specimen are shown in f) for the lens imaged in air and g) imaged in 4% BSA in PBS.

To confirm that the interference patterns observed in the images resulted from SW excitation and to verify that anti-nodal spacings and FWHMs were comparable to the theoretical values we imaged the fluorescently labelled lens specimens with different media between the specimen and the mirror (Fig. 2(b) and (d)

). Through taking a radial average for each image the average experimental anti-nodal spacings and FWHMs (Fig. 2(c) and (e)) were determined and compared to the theoretical values that were calculated from an analysis of the theoretical PSF calculated using Eq. (1). To determine if the theoretical and experimental values were statistically different we applied a single sample t-test compared to the theoretical values where P < 0.05 was considered significant. Results for these measurements can be seen in Table 1

**Table 1:** Comparison of experimentally determined anti-nodal spacings and FWHM obtained from lens specimen imaging and the theoretically determined values calculated using Eq. (1) where the peak excitation wavelengths was 548.8 nm, the peak emission wavelength was 570 nm.

**LED**	**Refractive index of media**	**Theoretical anti-nodal spacing (nm)**	**Experimental anti-nodal spacing (nm)**	**Statistical significance**	**Theoretical anti-nodal FWHM (nm)**	**Experimental anti-nodal FWHM (nm)**	**Statistical significance**
**550 nm**	1	273.8	275.0 ± 3.8	P > 0.05	136.9	134.0 ± 4.4	P > 0.05

	1.341	204.3	204.9 ± 3.0	P > 0.05	102.2	99.6 ± 1.2	P > 0.05

.

### 3.2 Video-rate SW imaging and computation reconstruction of red blood cells

**Fig. 3:**
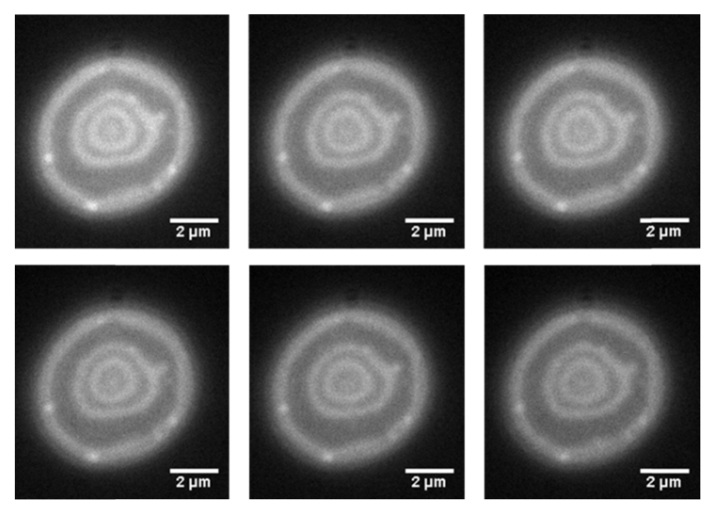
Single frames taken from the video-rate SW movie of the bottom half of a red blood cell labelled with the membrane dye DiI using a camera binning n = 2. The frames presented as a-f are frames 1, 200, 400, 600, 800 and 999.

Using the optical setup shown in Fig. 1 it was possible to carry out widefield SW imaging of the bottom half of a fluorescently labelled red blood cell at speeds in excess of video-rate (Fig. 3

). The optical attenuation by a single red cell has been measured previously to be around 20% in the green spectral region which we used to excite DiI [26], which supports the propagation of the excitation light and, following reflection from the mirror surface below the cell, allowing the formation of the standing wave. By carrying out this technique we were able to image rapid membrane fluctuations and changes in real time that would not be observable using point scanning methods due to the limited temporal resolution available. Using the data obtained from the resolution measurements and the imaging of the fluorescently coated lens specimens in 4% BSA in PBS we can conclude that we are achieving a lateral resolution of 290 nm and an axial resolution of 99.6 ± 1.2 nm. The full 1000 frame raw SW movie can be seen in the supplementary video, Visualization 1, which has a playback speed of 10 frames per second and has undergone JPEG compression.

**Fig. 4:**
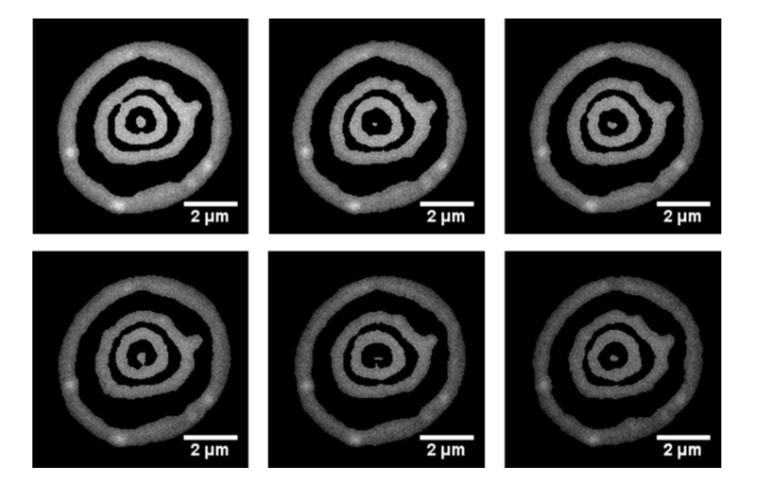
2D reconstruction of the SW movie with the original intensity values applied to the isolated anti-nodal planes. The frames presented as a-f are frames 1, 200, 400, 600, 800 and 999.

Using the steps described in section 2.5 we were able to isolate the anti-nodal planes from the background and create a 2D reconstruction of the SW movie by mapping the original intensities to the threshold images (Fig. 4

). This resulted in a video showing only the red blood cell anti-nodal planes.

**Fig. 5:**
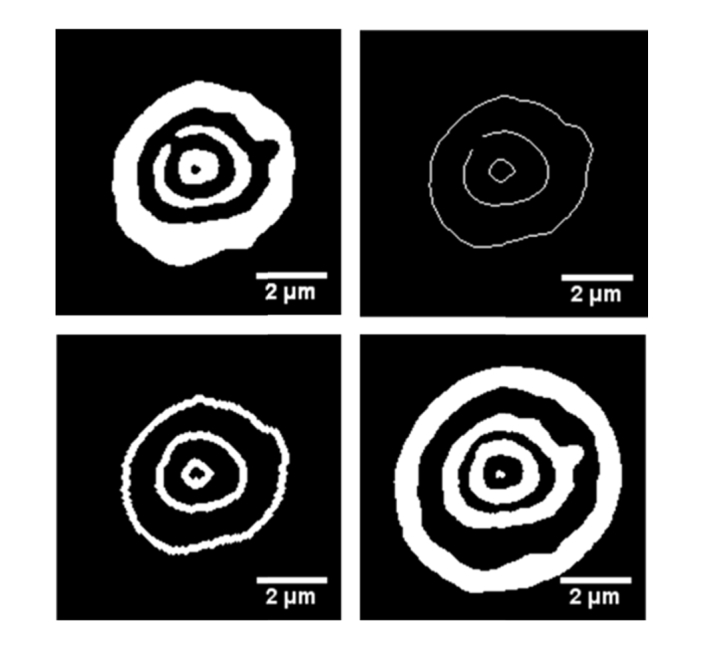
Key steps of the anti-nodal plane separation for frame 503 of the SW movie. a) Threshold image has been inversed and resulted in a black and white image of the nodal regions b) a skeletonized image of the nodal regions c) The resultant cubic interpolated nodal planes which have been dilated by three pixels. d) The anti-nodal planes have been separated by subtracting c) from the anti-nodal plane threshold image.

To create 3D reconstructions of the red blood cell membrane closest to the mirror, we then separated anti-nodal planes that appeared to be in contact with one another. The results of the key steps of the process described in section 2.5 for frame 503 of the SW movie can be seen in Fig. 5

.

**Fig. 6:**
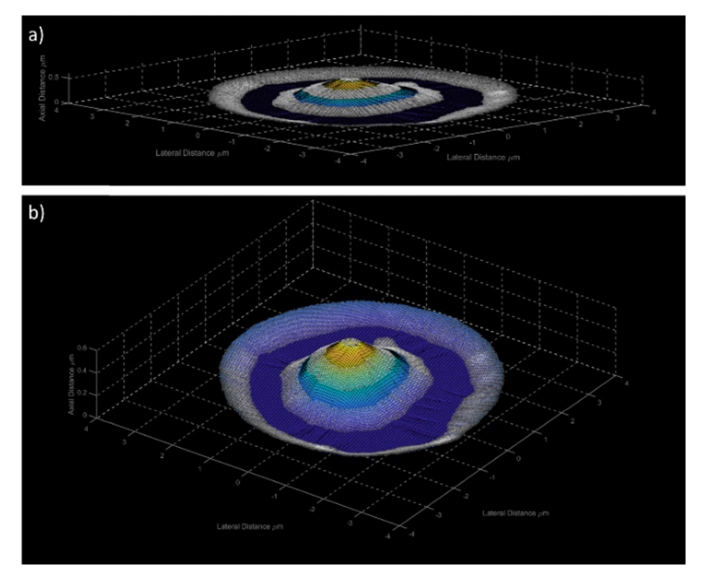
A 3D reconstruction the first frame of the SW movie where a) where the aspect ratio has not been altered and b) the aspect ratio of the z axis has been increased to 13.33 for easier visualization.

Using Eq. (3) and Eq. (4), the theoretical axial positions were applied to the segmented edge detection regions, we then interpolated each axial height for the pixels between each plane edge. The 3D surface and the anti-nodal plane intensities are shown in Fig. 6

which aids in our understanding of the 3D membrane structure of the specimen and allows for the observation of morphological changes in the lateral and axial directions in real time. The 3D reconstruction of the SW movie, containing 981 frames, can be seen in the supplementary video, Visualization 2. This movie has undergone JPEG compression and has a playback speed of 10 frames per second.

### 3.3 Comparison between video-rate SW microscopy and video-rate widefield epifluorescence microscopy

**Fig. 7:**
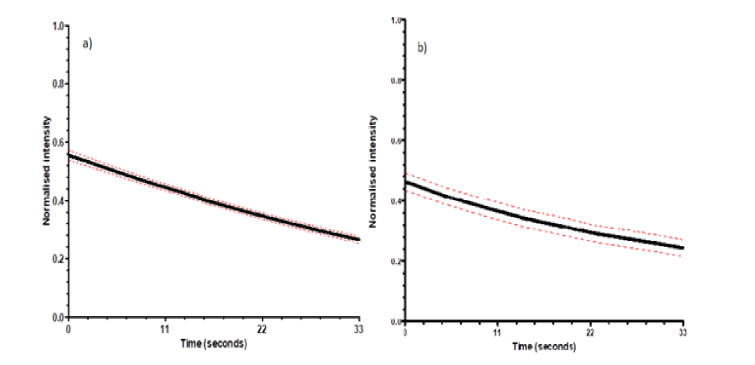
Average normalised intensity (solid line) and the standard error of the mean (red dashed line) from a) SW movies and b) widefield imaging of red blood cells (n = 10) excited using a 550 nm LED with an illumination power at the specimen plane of 1.71 ± 0.01 mW.

After successfully carrying out video-rate SW microscopy and generating 2D and 3D reconstructions of our red blood cell movies we investigated the effect of the SW imaging technique on the rate of specimen photobleaching and compare this to that recorded using video-rate widefield epifluorescence microscopy. We performed SW imaging of 10 red blood cells using the method described above. To investigate the photobleaching rates we inputted the cropped and contrast adjusted movies into the MATLAB script to obtain a normalized 2D reconstruction for each one. As before, the MATLAB script applied a local threshold to isolate the anti-nodal planes and we found the average intensity of all the non-zero intensity pixels and outputted this value for each time point. This process was repeated but with the red blood cells on standard microscope slides rather than a mirror to image the cells using standard widefield epifluorescence illumination rather than with a SW. The average normalized intensity along with the standard error of the mean for each time point (n = 10) for SW microscopy can be seen below in Fig. 7(a)

and for widefield epifluorescence imaging in Fig. 7(b).

When comparing the data using both techniques we measured that the average intensity decrease observed across the 1000 frames when using SW imaging was 51.89 ± 2.28% and for standard widefield epifluorescence imaging it was 47.78 ± 4.78%. We compared these rates using a student t-test after which it was found that there was no significant difference between the average photobleaching rates obtained with each technique (P > 0.05).

We also compared the average initial normalized intensity values when using each technique and found that the SW imaging provided an initial normalized intensity of 0.55 ± 0.02 which was significantly larger than that with widefield microscopy using the same power at the specimen plane (n = 10, P < 0.05 compared to an initial intensity using widefield imaging of 0.46 ± 0.03). We attribute the increased brightness of the SW images compared to the widefield epifluorescence images to be due to the multiple in-focus fluorescent planes being present in the SW images. In widefield epifluorescence microscopy there is only a single plane in focus with the rest of the image being composed of out of focus fluorescence.

**Fig. 8:**
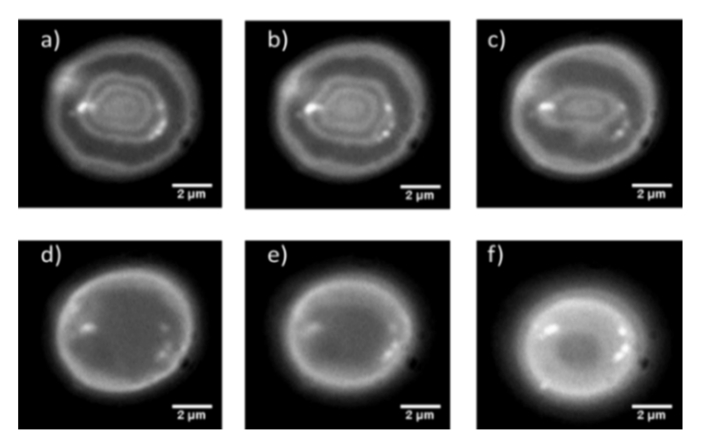
Cropped and contrast adjusted SW images of a red blood cell. The frames presented as a-f are at time points 15, 360, 720, 1080, 1440 and 1800 s.

**Fig. 9:**
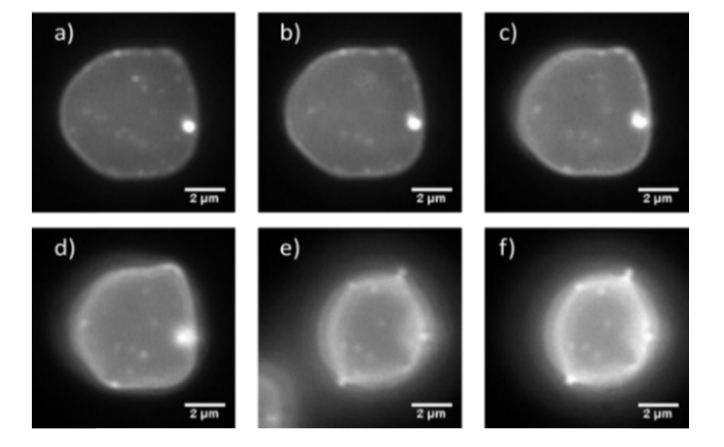
Cropped and contrast adjusted widefield epifluorescence images of a red blood cell. The frames presented as a-f are at time points 15, 360, 720, 1080, 1440 and 1800 s.

We also explored the effect of our imaging technique on red cells over longer timescales. We placed red cells on either a mirror or a microscope slide and imaged the specimens using an LED and camera exposure time of 100 ms and capture an image every 15 seconds for a duration of 30 minutes. Six time point images from these experiments can be seen in Fig. 8

for imaging with a SW and Fig. 9

for widefield epifluorescence imaging.

As can be seen from the above figures, SW microscopy reveals a greater amount of topographical information about the decay of the red blood cell. It is also apparent that both red blood cells appear to flatten and undergo membrane deformations at approximately the same rate regardless of which technique was used.

## 4. Discussion

All previous applications of SW microscopy have utilized lasers [10,13,15,27,28], for illumination, but in our research, we have utilized an LED source for illumination. LEDs are becoming an increasingly more common illumination source in many microscope labs because of narrow emission bandwidths across the entire visible electromagnetic spectrum, precise electronic intensity control, and rapid switching speeds.

As we believe this to be the first use of an LED illuminator for SW fluorescence microscopy, we sought to confirm that we were able to obtain experimental anti-nodal spacing and FWHMs that were comparable to the theoretical values. To do this we replicated the model fluorescent lens specimen imaging experiments of Amor et al. with media of different refractive indices between specimen and the mirror. It was found through these experiments that we were able to obtain, in all media, anti-nodal spacings and FWHMs that were not significantly different from the theoretical values (P > 0.05).

It has been reported previously that DiI is resistant to photobleaching [19–21], however, in our experiments we observed approximately a 50% intensity decrease over the 33 second imaging period. We attribute this to either the majority of the decay occurring during the specimen preparation and finding of the specimens prior to imaging. Another explanation is perhaps due to a contribution of a dye interaction with oxygen which has been observed to increase the photobleaching rates of DiI [29–31]. Photobleaching rates have always been a limiting factor when carrying out microscopy and can be especially problematic when carrying out super-resolution microscopy [32,33], so the ability to cause no increased rate in bleaching compared to widefield epifluorescence imaging can be a significant advantage.

We note that some other super-resolution microscopy techniques may require the use of specific dyes [33–36], whereas SW microscopy is compatible with standard fluorescent membrane dyes commonly employed in widefield epifluorescence microscopy. Another advantage of utilising SW microscopy is that it can be performed using a typical widefield epifluorescence microscope with the only change required for specimen preparation being the replacement of a microscope slide with a mirror, whereas some other super-resolution techniques may require the use of specialist microscopes [37–40]. This is also an advantage held over other imaging methods which has been used extensively for the study of red blood cell morphology [41–43], such as quantitative phase imaging or differential interference contrast microscopy, which requires the addition of polarizers or prisms into the optical path and as such may require modification to be carried out to an existing system or the purchase of special objective lenses [44–46].

Interference reflection microscopy (IRM) has also been used previously to image red blood cells allowing the specimens to be imaged without using a fluorescent probe, and results in images that resemble those obtained using our SW technique [47,48]. However, the Curtis model of IRM has been shown to be inconsistent with membrane topography measured by other methods. For example, Gingell, et al. [49] measured the thickness of the space between coverslip and the membrane of chick fibroblasts by including a cell impermeable fluorescent dye in the extracellular space and found by total internal reflection fluorescence microscopy (TIRF) excitation that there was close contact (shown by dye exclusion) over almost all the area of the cell facing the coverslip and not just in the 'close contact zones' or 'focal adhesions' seen in IRM. They also remarked that a dark peripheral band was seen in IRM of some cells, which had no counterpart in the TIRF image. They speculated that the IRM image was influenced not by the membrane topography but by the thickness of the cytoplasm in that region. More recently, Iwanaga et al. [50] used a standing-wave method similar to ours, in which the highly hydrophobic dye DiI was used to stain the cell membrane specifically, and, again, there was no evidence for the 'close contacts' that were interpretations of the dark zones in IRM. These authors raised the cell membrane ingeniously on silica microsteps away from the reflecting layer and showed that the contrast changes were as expected if the membranes followed the steps faithfully and were excited at the anti-nodal planes in the standing wave pattern, demonstrating that their method, which they called 'Fluorescence Interference Contrast' (but which was in fact a standing wave method like ours), was a reliable method for studying membrane topography, whereas IRM was not. Not only is IRM ambiguous, in that it cannot be assumed to report membrane position, but it is also affected by scatter so that absolute values for axial distances and resolutions cannot be obtained from the observed contrast in IRM images. The standing wave method provides an unequivocal axial ruler related to the wavelength and refractive index of the medium and, since it involves a specifically localized fluorophore, it reports the position of the membrane unaffected by cytoplasmic contents.

It is also common to find that super-resolution techniques typically sacrifice temporal resolution in order to increase spatial resolution [51,52], whereas we were able to improve the widefield axial resolution by a factor of approximately 8 and maintain high temporal resolution imaging of live cell specimens in 3D. This was not possible in the work carried out by Amor et al. as they utilised a scanning confocal microscope for imaging [15]. The ability to carry this out on a widefield epifluorescence microscope also allows for the possibility of multiple red blood cells to be investigated simultaneously which could be an advantage for diagnosis or studies requiring a high data throughput.

Many super-resolution microscopy techniques can provide a spatial resolution improvement both axially and laterally, something which is not possible in SW microscopy, so it is likely that there will be circumstances where SW microscopy is not a suitable technique, such as when very high lateral resolution is required. Even with this limitation though, we were able to carry out video-rate SW microscopy which allowed rapid membrane fluctuations and the morphology of red blood cells to be imaged which can be an advantage for researchers looking to investigate membrane flickering and the biomechanical characteristics of red blood cells [53–55], or the behaviour of diseased or healthy red blood cells undergoing morphological changes [2,56–59].

The flickering phenomenon observed in red blood cell membranes has been widely reported and was documented as early as 1890 [60]. It has been suggested that the process may be due to either thermal processes or dynamic remodelling of the cytoskeleton and active membrane mechanisms [53,54,61]. The high temporal resolution of our technique has allowed us to observe rapid membrane changes in real time which appears to indicate that the entire membrane is in motion with whole-cell vibrations being observed and small movements taking place at the concave surface. The entire cell motion may be due to whole cell Brownian motion, or weak electro-static interaction between the poly-L-lysine coating and the membrane, though the ability to observe whole cell movements with super-resolved axial sections demonstrates that our technique may also have applications in cell tracking or motility studies. The motion observed in the concave surface resembles the movements which occur at the boundary between the concave section and the outer membrane [61], though the majority of this boundary inhabits a nodal plane. We were unable to observe the very small membrane dimpling observed in other studies using quantitative phase imaging which appear to be on the order of approximately 40 nm [58], and is to be expected as they are not large enough to cross the boundaries between an anti-nodal or nodal plane and we are not able to axially resolve small movements (~100 nm) within planes.

The application of SW microscopy can also be limited when a complete topographical map of the specimen is required because of the contribution of nodal planes resulting in periodic bands of missing information. Our computational method extracts and segments 3D anti-nodal information, though this did require some modest assumptions to be made of the membrane morphology in the nodal regions. We also note that the SW fringes mirror-side of the cell are very good (flat and equi-distant), but that the apical surface of the cell is more problematic to image with this method because the quality of the SW fringes degrades away from the mirror surface.

In specific circumstances, the method used for anti-nodal separation was unsuccessful and fewer than the full 1000 frames could be reconstructed in 3D. This occurred when images contained bright spots between the anti-nodal planes, which we believe to be free dye clumps, so we were unable to assign axial heights to these objects. The other situation was one where any planes were broken, which often arose from either the incorrect interpolation of the nodal regions, or due to the lack of data points caused by multiple anti-nodal contacts.

Using the assumption that there were only ever three nodal planes, the script was capable of automatically removing images which had too few or too many object edges compared to number of assumed nodes. Any subsequent frames missed by the automated method had to be removed manually from the 3D reconstruction movie and in the data presented here this only amounted to three frames of 984.

Through the application of our method and subsequent removal of incorrectly-reconstructed frames we were able to generate movies that contained up to 981 frames from the raw SW movies. Using the computational technique described, we were capable of taking widefield SW movies acquired at 30.30 Hz and output 2D reconstructions in approximately 10 seconds for the full thousand frames and 3D reconstruction in approximately 10 minutes for 981 frames. The CPU RAM required was 64 GB, however, it could be possible to split the SW movies into a smaller subset of frames and process them individually to reduce memory consumption.

We also investigated whether the SW technique caused any further photo-toxicity to the specimen compared to widefield epifluorescence imaging. It was observed that when using either imaging method, the red blood cell appeared to decay at a similar rate and in a manner that resembled the decay observed previously over 12 minutes under periodic focused laser illumination [62]. In the study of Wong et. al [62], it was found that membrane damage occurred even at low laser powers as the result of reactive oxygen species being generated via photo-hemolysis. This process has been hypothesized to be due to interactions between the reactive oxygen species and the membrane proteins, band 3 and spectrin [62], which has also been reported in studies using widefield illumination [63,64]. It has also been found that when using multiphoton microscopy, red blood cell photodamage could be observed on timescales in the region of tens of seconds [65], however, when we carried out our video-rate experiments we observed no photo-toxicity effects over the 33 second duration.

## 5. Conclusions

In this work we present the first demonstration of widefield SW imaging of fluorescently labelled red blood cells with high temporal resolution. Through the simple method of placing our specimens upon a mirror we were able to achieve axial super-resolution imaging below 100 nm of the morphology and deformation of the red blood cell membrane in real time with no increase in photobleaching rates or cellular toxicity compared to video-rate widefield epifluorescence imaging. We also describe a new computational method for extracting data from SW movies or images in order to create 2D and 3D reconstructions of the specimen allowing easier visualization of the data and could allow further quantitative analysis to be carried out.
